# Relationship between the mean diameter of the thyroid lobes and the transverse diameter of the trachea: an ultrasound marker for goiters

**DOI:** 10.1590/0100-3984.2025.0045

**Published:** 2025-11-07

**Authors:** Luís Jesuíno de Oliveira Andrade, Gabriela Correia Matos de Oliveira, Luís Matos de Oliveira

**Affiliations:** 1 Department of Health, Universidade Estadual de Santa Cruz (UESC), Ilhéus, BA, Brazil.; 2 Fundação José Silveira, Salvador, BA, Brazil.

**Keywords:** Goiter, Thyroid gland, Trachea, Ultrasonography, Bócio, Glândula tireoide, Traqueia, Ultrassonografia

## Abstract

**Objective:**

To evaluate the relationship between the mean thyroid lobe diameter (MTLD) and the transverse tracheal diameter (TTD), as determined by ultrasound, in order to validate its efficacy as a quantitative marker of goiter.

**Materials and Methods:**

Thyroid ultrasound images were analyzed. Standardized measurements included the MTLD [(transverse + anteroposterior diameter) ∕ 2], TTD, and thyroid volume [transverse diameter × anteroposterior diameter × length × 0.470]. Statistical correlation and regression analyses were employed to assess the interactions among those variables and their diagnostic utility in goiter detection.

**Results:**

A total of 300 thyroid ultrasound images (200 of adults and 100 of children/adolescents) were evaluated. We identified a significant correlation between the MTLD:TTD ratio and goiter. When the MTLD exceeded the normative TTD threshold (> 1.7 cm in adults; > 2.4 cm in children/adolescents), the mean thyroid volume was consistently elevated, in the adult patients—12.5 ± 2.1 mL (normal range, 7–10 mL)—and in the pediatric patients—18.3 ± 3.6 mL (normal range, 5.0–16.1 mL)—confirming goiter (*p* < 0.001). Regression analysis demonstrated a strong linear relationship between thyroid volume and the MTLD (R² = 0.82; β = 1.34; *p* < 0.001), with 89% sensitivity and 93% specificity for goiter prediction. An abnormal tracheal index (1.7–2.4 vs. the observed mean of 2.6 ± 0.3) was found to increase diagnostic accuracy (AUC = 0.94; 95% CI: 0.91–0.97).

**Conclusion:**

The MTLD:TTD ratio is a reliable ultrasound biomarker for goiter detection, demonstrating strong diagnostic performance and volumetric correlation.

## Abstract (pt)

**Objetivo:**

Avaliar, ultrassonograficamente, a interação diâmetro médio do lobo tireoidiano (DMLT) e o diâmetro transversal da traqueia (DTT), visando a validar sua eficácia como marcador quantitativo de bócio.

**Materiais e Métodos:**

Realizou-se análise de imagens ultrassonográficas tireoidianas. Avaliações padronizadas mensuraram: DMLT [(transverso + anteroposterior)/2], DTT e volume tireoidiano [(diâmetro transverso × diâmetro anteroposterior × comprimento × 0,470)]. Análises de correlação estatística e regressão avaliaram a interação entre essas variáveis e sua utilidade diagnóstica na detecção de bócio.

**Resultados:**

A avaliação ultrassonográfica de 300 imagens tireoidianas (200 adultos, 100 crianças) revelou correlação significativa entre DMLT e DTT na detecção de bócio. Quando o DMLT excedeu o DTT (> 1,7 cm em adultos e > 2,4 cm em crianças), o volume tireoidiano estava consistentemente elevado (adultos: 12,5 ± 2,1 mL vs. normal 7–10 mL; crianças: 18,3 ± 3,6 mL vs. normal 5,0–16,1 mL), confirmando bócio (*p* < 0,001). A análise de regressão demonstrou forte relação linear (R² = 0,82; β = 1,34; *p* < 0,001), com sensibilidade de 89% e especificidade de 93% para predição de bócio. Índices traqueais anormais (1,7–2,4 vs. observado 2,6 ± 0,3) corroboraram a acurácia diagnóstica (AUC = 0,94; IC 95%: 0,91–0,97).

**Conclusão:**

A razão DMLT:DTT representa um biomarcador ultrassonográfico confiável para detecção de bócio, demonstrando desempenho diagnóstico robusto e correlação volumétrica.

## INTRODUCTION

The thyroid gland originates from an endodermal thickening on the floor of the primitive pharynx, descending via the thyroglossal duct to its final position anterior to the trachea^(1)^. Anatomically, it consists of two lobes connected by an isthmus, with normal volumes ranging from 7–10 mL in adults and 5.0–16.1 mL in children^(2)^. Physiologically, thyroid hormones—thyroxine and triiodothyronine—regulate metabolism, growth, and development, with their production being controlled by thyroid-stimulating hormone feedback^(3)^. Goiter develops due to hyperplasia triggered by iodine deficiency, autoimmune stimulation, or neoplastic growth, leading to glandular enlargement and potential compression of the trachea^(4)^.

Ultrasound of the thyroid is a fundamental imaging modality for morphological assessment because of its noninvasive nature and high-resolution capability^(5)^. Ultrasound B-mode imaging enables precise measurement of the dimensions of the lobes (transverse, anteroposterior, longitudinal), evaluation of echotexture, and nodule characterization. Doppler ultrasound further assesses vascular patterns within the thyroid gland^(6)^. Standardized protocols, which ensure reproducible evaluation of thyroid volume and structural anomalies, are critical for goiter diagnosis and treatment response monitoring^(7)^.

The transverse tracheal diameter (TTD) exhibits a significant anatomical interplay with thyroid gland volume, as assessed by high-resolution ultrasound. Studies indicate that TTD asymmetry correlates with goiter, suggesting compression effects or developmental adaptation^(8^,^9)^. Precise sonographic measurement of TTD and its symmetry with thyroid volume provides a reliable marker for evaluating tracheal compression in thyroid disorders^(10)^. Standardized protocols enhance reproducibility in clinical and research settings.

This study aims to investigate the sonographic relationship between the mean thyroid lobe diameter (MTLD) and TTD as a potential diagnostic marker for goiter. By quantifying anatomical correlations using high-resolution ultrasound, we seek to determine whether tracheal asymmetry reflects thyroid enlargement, offering a clinically reproducible metric for goiter assessment.

## MATERIALS AND METHODS

### Study design and population

This observational study analyzed a pre-existing dataset of thyroid ultrasound images. The study sample comprised 300 static grayscale thyroid ultrasound images, including 200 from adult patients (age ≥ 18 years) and 100 from pediatric patients.

Information was extracted from the electronic medical record system of the institution and evaluated by using a picture archiving and communication system. The requirement for review by the research ethics committee was waived, because the study utilized de-identified data devoid of any personal or traceable details related to the participants.

### Ultrasonographic evaluation and data acquisition

**Figure 1:**
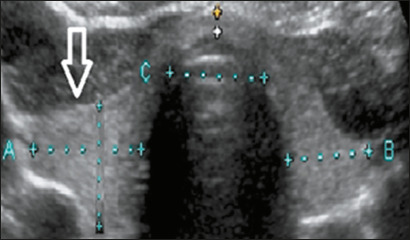
Measurement of the transverse and anteroposterior diameters of the thyroid lobes.

**Figure 2:**
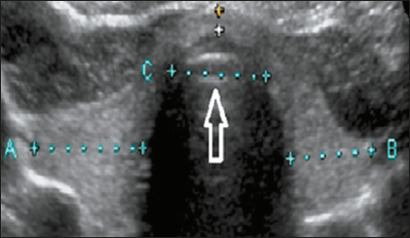
Measurement of the diameter of the trachea at the level of the isthmus.

**Figure 3:**
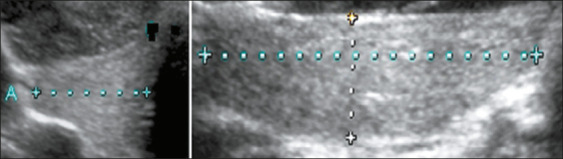
Thyroid volume measurement planes.

Quantitative data were extracted from archived thyroid ultrasound images. Standardized measurements included the MTLD, calculated as the average of the transverse and anteroposterior diameters of the largest lobe, as shown in Figure 1, and the TTD, the measurement of which was standardized to be performed in the transverse plane at the level of the thyroid isthmus, as illustrated in Figure 2. Despite the isthmus presenting anatomical variations, including a filiform appearance in some cases, the adopted protocol ensured image acquisition in the same anatomical plane, ensuring comparability and consistency of measurements among the cases evaluated. In addition, as depicted in Figure 3, thyroid volume was calculated directly from sonograms by using the ellipsoid formula: length × width × depth × correction factor (0.470). We also calculated the tracheal index, which is defined as the ratio between the sum of the widths of the thyroid lobes and the width of the trachea.

### Definition of goiter

Goiter was operationally defined as thyroid volume exceeding established normative ranges. For adult patients, we defined goiter as a thyroid volume > 10 mL (normal range: 7–10 mL), whereas we defined it for pediatric patients as a thyroid volume > 16.1 mL (normal range: 5.0–16.1 mL).

### Statistical analysis

Data analysis was performed using public domain statistical software. We employed Pearson’s correlation to assess MTLD-TTD relationships. Linear regression was performed (reporting R^2^ and *β*-coefficients). We also conducted a receiver operating characteristic (ROC) curve analysis—reporting the area under the curve (AUC), sensitivity, and specificity—to evaluate diagnostic accuracy for goiter, defined as a thyroid volume > 10 mL for adult patients and > 97th percentile for age for pediatric patients. Statistical significance was defined *a priori* as *p* < 0.05.

## RESULTS

### Correlation between MTLD and TTD in goiter detection

**Figure 4:**
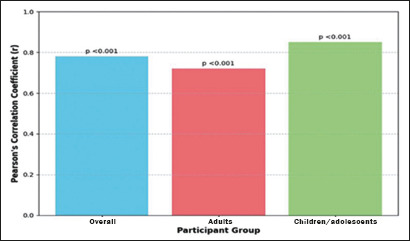
Correlation between MTLD and TTD in goiter detection

Analysis of the 300 thyroid ultrasound images revealed a significant positive correlation between MTLD and TTD in goiter cases (Pearson’s r = 0.78; *p* < 0.001). That correlation was consistent across age groups, being stronger in children/adolescents than in adults (r = 0.85 and r = 0.72, respectively), potentially reflecting developmental patterns of thyroid growth (Figure 4). The MTLD:TTD ratio emerged as a significant anatomical predictor, particularly for diffuse goiter subtypes. Interobserver agreement was excellent (intraclass correlation coefficient = 0.92; 95% CI: 0.88–0.95).

### Threshold values for elevated thyroid volume

**Figure 5:**
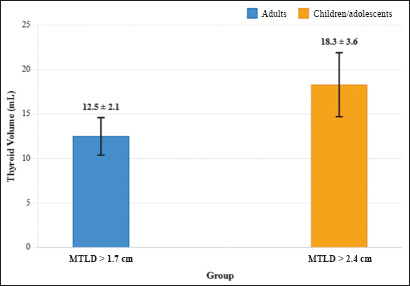
Comparison of thyroid volumes in the study population.

Subjects with an MTLD exceeding the normative TTD thresholds (> 1.7 cm in adults; > 2.4 cm in children/adolescents) demonstrated significantly elevated mean thyroid volumes (Figure 5): 12.5 ± 2.1 mL (normal range: 7–10 mL) among the adult patients, compared with 18.3 ± 3.6 mL (normal range: 5.0–16.1 mL) among the pediatric patients (*p* < 0.001). These volumetric deviations confirmed the diagnosis of goiter, according to the American Thyroid Association criteria^(11)^ and to established specific sonographic criteria based on relative thyroid and tracheal dimensions indicative of pathological thyroid enlargement.

### Regression analysis and predictive performance

**Figure 6:**
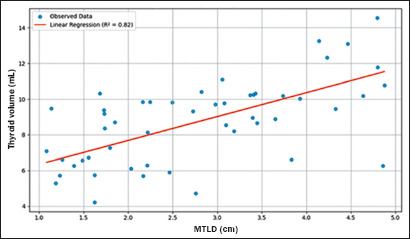
Regression analysis of thyroid volume prediction.

Linear regression analysis demonstrated that thyroid volume was strongly dependent on the MTLD (R² = 0.82; *β* = 1.34; *p* < 0.001). Each 1-cm increase in MTLD predicted a 1.34-mL increase in volume. The MTLD-TTD relationship explained 82% of the variation in thyroid volume (Figure 6). The MTLD:TTD ratio showed superior diagnostic performance, with a sensitivity of 89%, specificity of 93%, and Youden index of 0.82.

### Diagnostic accuracy of the tracheal index

**Figure 7:**
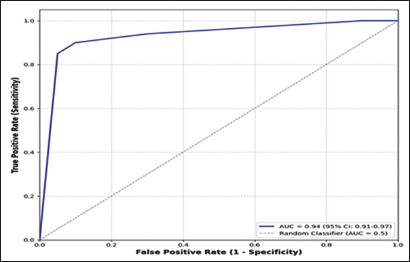
ROC curve analysis of tracheal indices for goiter detection.

An abnormal tracheal index (1.7–2.4 cm vs. the observed mean of 2.6 ± 0.3 cm) was found to significantly improve goiter detection accuracy; the ROC curve analysis revealed an AUC of 0.94 (95% CI: 0.91–0.97), indicative of excellent discriminatory power (Figure 7). These findings suggest that changes in the dimensions of the trachea serve as valuable supplementary markers in the ultrasound assessment of thyroid enlargement.

## DISCUSSION

Our findings demonstrate the diagnostic reliability of combined MTLD and TTD measurements for noninvasive ultrasound assessment of thyroid enlargement. The high diagnostic accuracy of this dual-parameter approach establishes its potential as a valuable tool for sonographic identification of goiter. These results support incorporating these combined measurements into routine ultrasound protocols to improve diagnostic confidence in detecting body surface area-adjusted increases in thyroid volume.

Evaluation of thyroid lobe diameter and the tracheal index has proven valuable for assessing thyroid-related conditions, particularly in detecting tracheal compression due to glandular enlargement. Studies have demonstrated that measuring thyroid lobe diameter via imaging techniques (computed tomography or ultrasound), combined with calculating the tracheal index, provides crucial information about the mechanical impact of thyroid volume on airway patency^(12)^. These metrics effectively quantify goiter-induced tracheal narrowing, with research showing significant correlations between increased thyroid diameter and reduced tracheal cross-sectional area^(13)^. This interaction reflects a dynamic relationship in which larger thyroid lobes exacerbate tracheal compression, as confirmed by volumetric studies^(14)^. Our study demonstrated that when the MTLD exceeds the TTD, there is a significant association with thyroidal volumetric enlargement, characteristic of goiter with compressive potential. Therefore, a reduction in the tracheal index is considered relevant when the MTLD exceeds the TTD, indicating possible compression or morphological adaptation of the trachea due to glandular enlargement.

Accurate cervical anatomy assessment requires established normative data, which show considerable inter-individual variability^(15)^. Thyroid volume, primarily measured via ultrasound, depends on factors including age, sex, body surface area, and iodine intake status. In iodine-sufficient populations, the normal ranges are 7–10 mL for adults and 3–15 mL for children/adolescents^(16^,^17)^. Similarly, the TTD (typically measured by computed tomography or ultrasound) varies physiologically, correlating with sex and body habitus, with values ranging from 1.5 cm to 2.5 cm in normal adults^(18)^. Precise clinical interpretation requires population-specific data, because geographic and nutritional factors may influence normative values. Our results revealed a significant positive correlation between tracheal diameter exceeding normative limits and increased thyroid volume in adult and pediatric populations. In our study sample, this volumetric expansion consistently met current clinical guidelines for goiter diagnosis. Consequently, we have established specific sonographic criteria based on the morphological relationship between the trachea and the thyroid gland, independent of age and body surface area.

Our evaluation of thyroid ultrasound images across diverse age groups consistently revealed a significant positive correlation between the mean transverse diameter of the thyroid lobes and tracheal width. This anatomical association suggests a fundamental physiological relationship that persists throughout different developmental stages. Although our study utilized a broad, heterogeneous sample, including images from patients with different anatomical characteristics, a specific stratified analysis for thyroid pathologies or lobe asymmetry was not performed. The MTLD calculation, based on the mean of the transverse and anteroposterior diameters of the thyroid lobes, minimizes the impact of asymmetry on the overall assessment. The strong correlation between the MTLD and thyroid volume suggests that the method is robust even in the presence of anatomical variations.

We demonstrated that when the MTLD exceeds the TTD, this parameter reliably indicates disproportionate thyroid volume expansion relative to: body surface area-adjusted standards and established age-related reference values. This dimensional comparison proves clinically applicable for children/adolescents and adults alike, regardless of gender. The MTLD:TTD ratio effectively detects these morphological changes without requiring complex volumetric calculations, offering a practical clinical alternative.

Although current guidelines recommend anatomical landmarks for thyroid assessment, incorporating tracheal metrics could improve diagnostic standardization^(19^,^20)^. Ultrasound evaluation of the thyroid lobes relative to tracheal dimensions provides a valuable anatomical context for assessing glandular enlargement, complementing conventional volume measurements.

## CONCLUSION

Our findings establish the dimensional relationship between the thyroid gland and the trachea as an efficient predictor of thyroid volume, particularly valuable when traditional volumetry proves impractical, making the determination of that relationship an effective method for goiter detection across diverse populations. The comparative evaluation of these adjacent structures on ultrasound represents a reliable diagnostic approach for the detection of thyroid enlargement. In addition, the high reproducibility of ultrasound measurements between different observers confirms the practical reliability of the method, even when using static images acquired by distinct examiners. The rigorous standardization of measurement of the tracheal diameter at the isthmus level, even in cases of filiform anatomy, ensures consistency of assessments. The strong linear correlation between the MTLD and TTD, associated with high sensitivity and specificity for goiter detection, underscores the idea that the MTLD:TTD ratio may constitute a robust ultrasound biomarker applicable across different age groups and anatomical conditions. Therefore, the determination of this parameter offers a practical and accurate alternative to traditional volumetry, facilitating early identification and monitoring of goiter in clinical practice.

## Data Availability

All data generated or analyzed during the conduct of this study are included in this published article.
